# Type-I interferon response affects an inoculation dose-independent mortality in mice following Japanese encephalitis virus infection

**DOI:** 10.1186/1743-422X-11-105

**Published:** 2014-06-05

**Authors:** Kotaro Aoki, Satoshi Shimada, Dash Sima Simantini, Mya Myat Ngwe Tun, Corazon C Buerano, Kouichi Morita, Daisuke Hayasaka

**Affiliations:** 1 Department of Virology, Institute of Tropical Medicine, GCOE program, Leading Graduate School Program, Nagasaki University, 1-12-4 Sakamoto, Nagasaki 852-8523, Japan

**Keywords:** Japanese encephalitis virus, Type-I interferon, Mouse model, Inoculation dose-independent mortality

## Abstract

**Background:**

The laboratory mouse model is commonly employed to study the pathogenesis of encephalitic flaviviruses such as Japanese encephalitis virus (JEV). However, it is known that some strains of these viruses do not elicit a typical mortality dose response curve from this organism after peripheral infection and the reason for it has not yet been fully understood. It is suggested that induction of more vigorous Type-I IFN (IFN-I) response might control early virus dissemination following increasing infectious challenge doses of the virus. Thus, the objective of this study was to examine this suggested role of IFN-I in the mortality of mice infected with various doses of JEV.

**Methods:**

Inbred 129 mice and their IFNAR KO (A129) mice were subcutaneously inoculated with 10^0^, 10^2^, 10^4^ or 10^6^ pfu of JaOArS982 strain of JEV. Mice were weighed daily and observed for clinical signs. Virus titers in the brains and spleens of JEV-infected mice were determined by plaque forming assays. The upregulated mRNA levels of genes related to IFN-I response of mice were examined by real-time PCR.

**Results:**

The mortality rates of 129 mice infected with JaOArS982 did not significantly increase despite the increase in inoculation dose and no significant difference of viral loads was observed between their brains. However, there was clear elevation of the mRNA levels of interferon regulatory factor (IRF)3, IRF7, IRF9, MDA5 and RIG-I at 24 hours post-infection depending on the inoculation dose. In A129 mice, length of survival days and the viral loads of spleen and brain were observed to be inoculation dose-dependent.

**Conclusions:**

From these results, it is suggested that early IFN-I response elicited by high inoculation doses of JEV provides an anti-viral effect during the early phase of infection. Accordingly, virus replication is counteracted by IFN-I response at each increasing inoculation dose resulting in the interference of impending severe disease course or fatal outcome; hence, this might explain the inoculation dose-independent mortality in mice caused by Japanese encephalitis virus.

## Background

Japanese encephalitis virus (JEV) belonging to the genus *Flavivirus* of the family *Flaviviridae*, is a causative agent of Japanese encephalitis (JE), an acute central nervous system (CNS) disease in humans [1]. JE is considered as one of the most important encephalitic arthropod-borne diseases. An estimated 3 billion people live in countries where JE is endemic and 30,000 - 50,000 cases and 10,000 - 15,000 deaths are reported annually [1-3]. However, because many cases in less well developed countries are almost certainly unreported, this is likely to be a gross underestimate of the actual number of cases that either result in fatality or infection with severe sequelae. Thus, it is important to understand the mechanism of the development of the disease especially in severe cases.

To study the CNS pathology induced by encephalitic flaviviruses such as JEV and tick-borne encephalitis virus (TBEV), the laboratory mouse model is commonly employed. The reason is that the pathologic changes observed in infected mouse brains are similar to those observed in humans [4-10].

In general, to evaluate the virulence and pathogenicity of virus infection in a mouse model, lethal dose has been used as an index and it is believed that an increase in inoculation dose can cause high mortality. However, it is known that mice infected peripherally with some strains of encephalitic flaviviruses do not exhibit a typical mortality dose response curve. Although this has been reported since the 1940’s [11], the reason for this apparent discrepancies has until now not been fully understood. We previously reported that late death following TBEV and JEV infections appears to be a key feature of inoculation dose-independent mortality [12,13]. Late death was observed in mice subcutaneously inoculated with 10^0^ to 10^6^ pfu of these viruses [12,13]. However, we were not able to fully elucidate why no significant difference was found between any of the mortality rates despite the increase in inoculation doses.

Recently, it was suggested that induction of more vigorous innate immune response might control early virus dissemination following increasing infectious challenge doses of the virus [8,14,15]. Thus, in this study, we focused on Type-I IFN (IFN-I) response induced at early phase following extraneural infection and examined its role in the mortality of mice.

## Results

### Inoculation dose-independent mortality in inbred 129 mice subcutaneously infected with JEV

**Figure 1:**
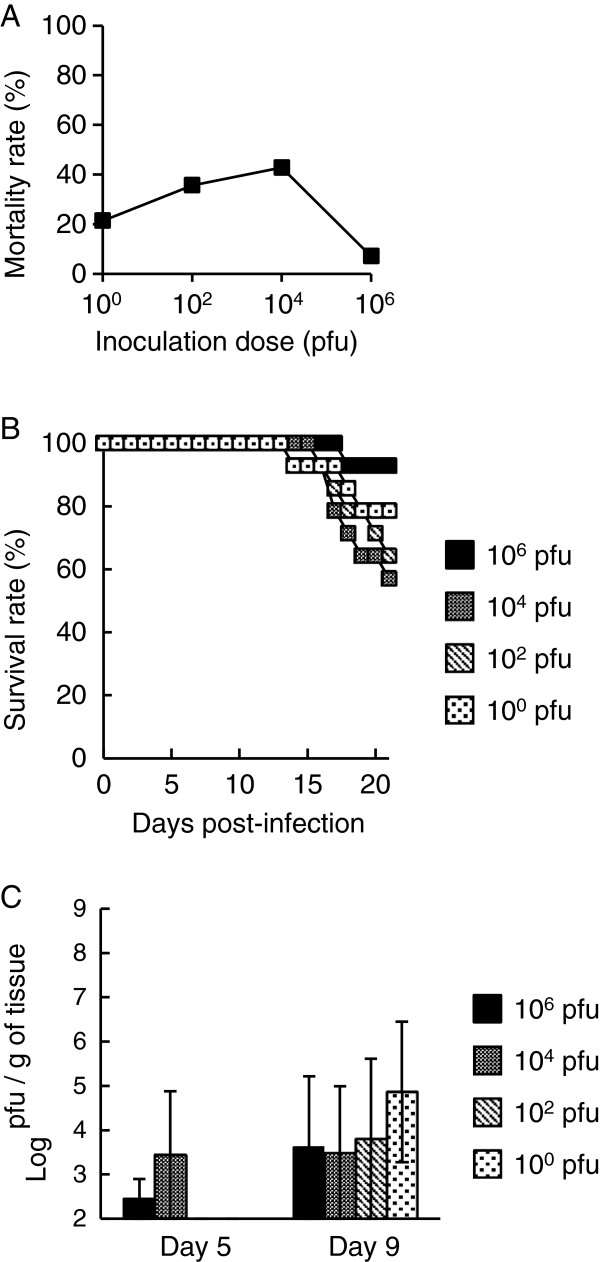
**Mortality and viral loads of 129 mice subcutaneously inoculated with the JaOArS982 strain of JEV.** Mortality **(A)** and survival **(B)** rates of mice inoculated with 10^0^, 10^2^, 10^4^ or 10^6^ pfu of JEV (n=14 per inoculation dose). Mortality rate was recorded 21 days pi. **(C)** Infectious virus titers in the brains of mice inoculated with 10^0^, 10^2^, 10^4^ or 10^6^ pfu of the JaOArS982 strain of JEV at 5 and 9 days pi (n=3 per inoculation dose and day of sacrifice). Error bars represent the standard errors.

In this study, we used inbred 129 mice to minimize the influence of the genetic background in each organism. Following subcutaneous inoculation with JaOArS982 strain of JEV, the mortality rates of infected mice did not increase significantly with increase in the inoculation dose (Figure 1A). Survival curves also did not differ significantly despite the increase in inoculation dose (Figure 1B). Mice that died exhibited general clinical signs such as slow movement, ataxia, piloerection, anorexia and continuous weight loss after 7–10 days post-infection (pi), but apparent clinical signs of CNS diseases such as paralysis were not observed as in our previous study [13]. The patterns of weight loss and clinical signs were also not significantly different at any time point between different inoculation doses (data not shown).Infectious virus was detectable in the brain tissues at 5 days pi (Figure 1C) but not in the peripheral tissues such as spleen (data not shown). Of note, at 5 and 9 days pi, no significant difference of viral loads was observed between brains from mice that received different doses of the virus (Figure 1C). These observations suggest that in this mouse model, mortality is not dependent on the inoculation dose, and that virus infection and replication seems to be being interfered at early phase before CNS infection.

### IFN-I response in the spleen of 129 mice infected with various doses of JEV

To examine IFN-I response, we first measured the levels of IFN-α and IFN-β in the serum using some commercial ELISA kits. However, these cytokines were not detected in all mice infected with various doses of JEV nor in all uninfected mice (data not shown). In the spleen, we also tried to detect and compare the expression levels of IFNs and IFN-I related proteins by Western blot, however, we failed. Thus, we next examined the mRNA levels of genes related to IFN-I response of mice by real-time PCR referred to in previous studies [12,13,16,17].

**Figure 2:**
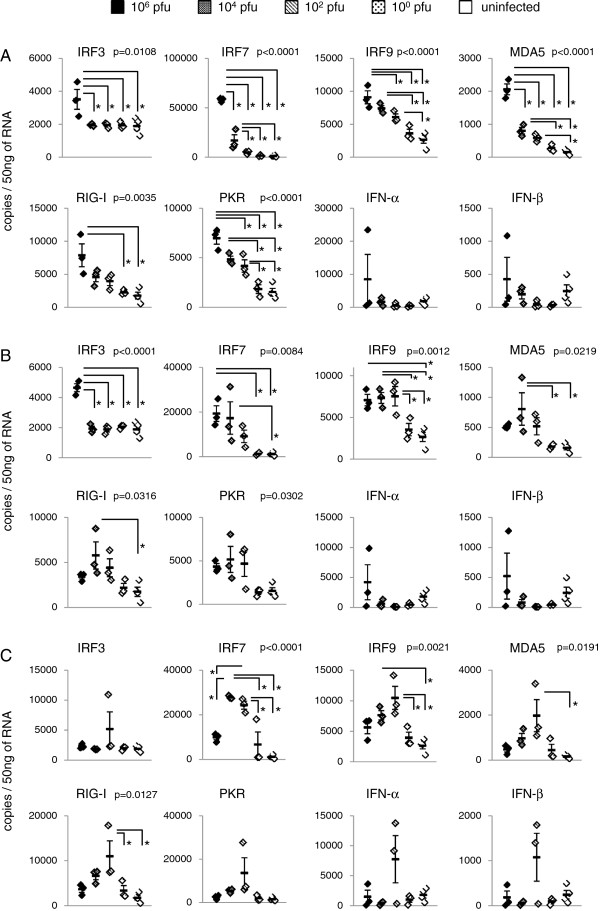
**mRNA levels of IFN-I related genes of 129 mice subcutaneously inoculated with the JaOArS982 strain of JEV.** mRNA levels were quantified by real-time PCR in the spleens of 129 mice inoculated with 10^0^, 10^2^, 10^4^ or 10^6^ pfu of JEV at 24 **(A)**, 48 **(B)** 72 **(C)** hours pi and of uninfected 129 mice (n=3 per group). Uninfected mice show the same data in **A**, **B**, and **C**. Error bars represent the standard errors. P values were calculated by ANOVA. Asterisk indicates the pair that shows significant difference by Tukey’s Multiple Comparison Test.

At 24 hours pi, the mRNA levels of interferon regulatory factor (IRF)3, IRF7, IRF9, MDA5 and RIG-I were clearly elevated depending on the inoculation dose of JaOArS982 with 10^6^ pfu per inoculation as the highest dose (Figure 2A). The mRNA level of PKR, one of ISG products induced by IFNs, was also increased as the viral dose was increased (Figure 2A). IFN-α and IFN-β also tended to elevate in an inoculation dose-dependent manner, although uninfected mice also exhibited some upregulation of these IFNs but the differences in the mRNA levels were not significant (Figure 2A).

At 48 hours pi, the mRNA levels of IRF3 and IRF7 were elevated depending on the inoculation dose (Figure 2B). However, the highest levels of MDA5, RIG-I and PKR were found in 10^4^ pfu-inoculated mice. IRF9 of 10^6^, 10^4^ and 10^2^ pfu-inoculated mice showed similar up-regulated levels (Figure 2B). IFN-α and IFN-β also tended to elevate in an inoculation dose-dependent manner, although the differences in the mRNA levels were not significant (Figure 2B).

At 72 hours pi, the level of IRF7 was higher in 10^4^and 10^2^ pfu-inoculated mice compared with other mice groups (Figure 2C). IRF9, MDA5 and RIG-I showed highest levels in 10^2^ pfu-inoculated mice compared with other mice groups (Figure 2C). IRF3, PKR, IFN-α and IFN-β also tended to be more elevated in 10^2^ pfu-inoculated mice, although the differences in the mRNA levels were not significant (Figure 2C).

These results suggested that the mRNA levels of IFN-I related genes can elevate as the dose of inoculated JEV is increased and that the peaks shift to lower inoculation doses as time passes by.

### Inoculation dose-dependent mortality in IFNAR KO mice infected with JEV

**Figure 3:**
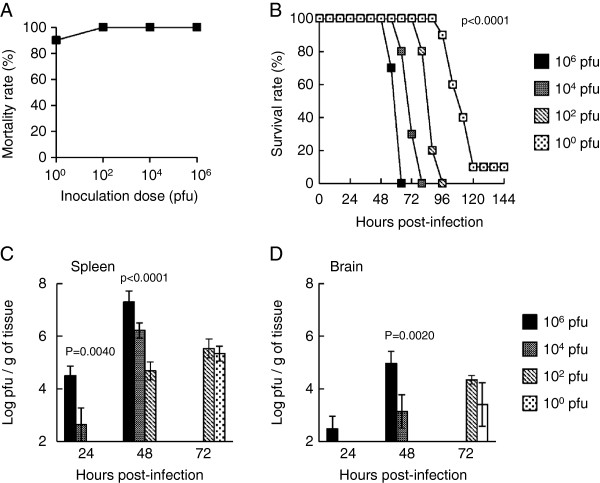
**Mortality and viral loads of A129 mice subcutaneously inoculated with the JaOArS982 strain of JEV.** Mortality **(A)** and survival **(B)** rates of mice inoculated with 10^0^, 10^2^, 10^4^ or 10^6^ pfu of JEV (n=10 per group). p: Log-lank test. Infectious virus titers in the spleens **(C)** and brains **(D)** of mice inoculated with 10^0^, 10^2^, 10^4^ or 10^6^ pfu of the JaOArS982 strain of JEV at 24, 48 and 72 hours pi (n=3 per group). Error bars represent the standard errors. P values were calculated by ANOVA at each time point.

To confirm whether IFN-I response is related to the dose-independent mortality, we next inoculated subcutaneously different doses of JaOArS982 in IFNAR KO (A129) mice and observed their mortality. Although a total of 90% (10^0^ pfu) or 100% (10^2^, 10^4^ and 10^6^ pfu) of JaOArS982-inoculated mice died during the observation period (Figure 3A), the length of survival hours were clearly inoculation dose-dependent (Figure 3B). Mice died by 64, 80, 96 and 120 hours pi following 10^6^, 10^4^, 10^2^ and10^0^ pfu inoculation doses, respectively (Figure 3B).

In the spleens, infectious viruses were detected initially at 24 h pi in 10^6^ and 10^4^ pfu-inoculated mice, at 48 h in 10^2^ pfu-inoculated mice, and at 72 h pi in 10^0^ pfu-inoculated mice, and the viral loads changed in a dose-dependent manner through time (Figure 3C). In the brains, infectious viruses were detected initially at 24 h pi in 10^6^ pfu-inoculated mice, at 48 h pi in 10^4^ pfu-inoculated mice, and at 72 h pi in 10^2^ and 10^0^ pfu-inoculated mice (Figure 3D). Thus, virus infection and replication in the peripheral tissues and brains are clearly inoculation dose-dependent in A129 mice.

## Discussion

In this study, we confirmed that mouse mortality is not dependent on the inoculation dose of JEV, that the increase in the mRNA levels of IFN-I related genes in mouse is suggested to be related to the increase of the dose of inoculated JEV, and that when IFN receptor is incapacitated during infection an inoculation dose-dependent mortality can occur in a mouse. Taken together, these suggest that IFN-I response affects the dose-independent mortality in a mouse model.

In our preliminary experiments, we intravenously injected constant amount of Poly (I:C) (a potent IFN inducer) or exogenous IFNs in mice following JEV infection to examine whether this treatment could provide protection in JEV-infected mice at lower inoculation dose but not at a higher dose. However, apparent protective effect on mortality by this treatment was not observed, and hence dose-independent mortality was not restored (data not shown). It could be due to technical problem that prevented IFN effects to reach local sites of infected tissues, because apparent IFN-I induction was not confirmed in the serum of mice injected with either inoculum (data not shown). However, this kind of approach is important because it may be able to give certain clues for elucidating further the mechanism on dose-independent mortality and thus further improvement of experimental design is required.

IFN-I response of JEV infected mice was initially examined by determining the levels of IFN-α and IFN-β in the serum through ELISA, but our attempt failed even in the mice that showed high mRNA levels of IFN-α and IFN-β in the spleen, e.g. those that received 10^6^ pfu inoculation at 24 hours pi and 10^2^ pfu inoculation at 72 hours pi. It could be due to technical difficulty. Therefore, the mRNA levels which were easier to detect by quantitative real-time RT-PCR were determined instead.

We examined the levels for IRF3, IRF7, IRF9, MDA5, RIG-I and PKR. IRFs play central roles in the induction of IFN-I at the gene transcriptional level [18]. IRF3 and IRF7 have been implicated as positive regulators of IFN-I gene expression induced by virus infections [18,19], whereas IRF9 constitutes an IFN-stimulated gene factor 3 together with STAT1 and STAT2, and is responsible for the induction of the IRF7 gene [18]. MDA5 and RIG-I function as cytoplasmic sensors of pathogen-associated molecular patterns within viral RNA and their expression is greatly increased with IFN-I exposure following virus infection [20]. They trigger the signal pathway of IRF3 and IRF7 [18,19]. PKR, an IFN-inducible gene product, binds to viral double-stranded RNA and halts protein synthesis by phosphorylating translation initiation factor eIF2 [21]. It plays an important role for the IFN-I induction, and its activation accompanies IRF3 activation [22,23]. The upregulation of the mRNA of these IFN-I related genes were observed in the present study in JEV infected mice and these reflected IFN response. A component or components of this response could have been affected following JEV infection at very high dose leading to a dose independent mortality.

Interestingly, it was observed that mRNA levels of IFN-α and IFN-β in the spleen of uninfected mice were somehow higher than those of 10^2^ and 10^0^ pfu-inoculated mice at 24 and 48 hours pi (Figure 2). Although One-way analysis of variance and Tukey’s Multiple Comparison Test used in this study showed no significant differences between them, these observations raised the possibility that low-dose inoculation with JEV might induce suppressive effects on IFN-I mRNA levels at early phase of infection. Further investigations will be required to elucidate this phenomenon.

In our previous and preliminary studies, we tried to detect mRNA of inflammatory genes including IFNs and their associated genes in the brains of JEV-infected mice. However, these mRNA were detected only after 5 days pi and the levels were not significantly different between mice inoculated with different doses. These observations showed patterns of viral loads similar to those shown in Figure 1. Clinical signs in fatal cases were observed after 7–10 days pi, but apparent CNS disease such as paralysis was not exhibited and their clinical signs (e.g. weight loss) were not significantly different between various inoculation doses. In our JEV-infected mouse model, main pathological changes and neuronal damage were observed in brain cortex [13]. The lesions seem to be related to memory deficiency and mental retardation but not paralysis and movement disorder. Thus, it was quite difficult to observe the CNS signs in JEV-infected mice, although lethal encephalitis was observed in dead mice. Encephalitis was a result of neuronal infection and subsequent inflammatory response. Systemic IFN-I response at early phase of infection appears to affect to viral CNS entry and neuronal infection. Therefore, we suggest that interference of inoculation dose-dependence by IFNs occurred in peripheral tissues, and thus subsequent neuronal infection and inflammatory responses including IFNs in the brains were not different between various inoculation doses.

We previously suggested that immunopathogenic responses in addition to high CNS infection contribute to the severe prognoses and we observed variable immune response in individual mouse infected with JEV or TBEV [13,24]. These data raise the possibility that there may be a variety of acquired immune response, e. g. specific T cell clones affecting either protective or pathogenetic functions in dying and recovering mice. Furthermore, we propose that the mortality following extraneural infection in mice does not simply represent neuroinvasiveness and thus it is difficult to compare pathogenesis by the lethal doses after peripheral inoculation in mouse model. To understand the pathogenic mechanism of flavivirus encephalitis, further elucidation of IFN-response, immunopathological effect, and their correlation will be an important priority to develop effective treatment strategies for flavivirus encephalitis.

## Conclusion

In conclusion, it is suggested that early IFN-I response of normal mice after receiving high inoculation doses of JEV provides an anti-viral effect during the early phase of infection. Virus replication is counteracted by IFN-I response at each increasing inoculation dose resulting in the interference of impending severe disease course or fatal outcome and this might explain the inoculation dose-independent mortality in mice caused by Japanese encephalitis virus.

## Methods

### Virus and cells

Stocks of JaOArS982 strain of JEV were obtained from cell culture medium of infected BHK cells [25]. The BHK cells were maintained in Eagle’s Minimal Essential Medium (EMEM) containing 10% fetal calf serum.

### Mice

Inbred 129 mice were provided by RIKEN BRC through the National Bio-Resource Project of the MEXT, Japan. A129 mice were purchased from B & K Universal limited. These mice were mated in the facility of Nagasaki University. Five to six week old mice [129 (n=14) and A129 (n=10)] were subcutaneously inoculated with 10^0^, 10^2^, 10^4^ or 10^6^ pfu of JaOArS982. Mice were weighed daily and observed for clinical signs for 21 days. The experimental protocols were approved by the Animal Care and Use Committee of the Nagasaki University (approval number: 091130-2-7 / 0912080807–7).

### Titration of virus in tissues

Mice subcutaneously inoculated with 10^0^, 10^2^, 10^4^ or 10^6^ pfu of JaOArS982 (n=3 for each dose) were euthanized on days 5 and 9 pi, and spleens and brain cortices were collected. Individual organ was homogenized and virus titers (expressed as pfu/g tissue) were determined by plaque forming assay in BHK cells [12,13].

### Quantitative estimation of the upregulation of IFN-I related genes in spleens

Mice subcutaneously inoculated with 10^0^, 10^2^, 10^4^ or 10^6^ pfu of JaOArS982 were euthanized, and spleens were collected. Experiments were carried out three times and data presented were pool of these experiments (thus a total of n=3 per dose). Total RNA was extracted and the mRNA levels of IRF3, IRF7, IRF9, MDA5, RIG-I, PKR, IFN-α and IFN-β were measured by real time-PCR as demonstrated previously [16].

### Statistical analyses

Statistical analyses were performed by using GraphPad Prism 5 (GraphPad Software, Inc). One-way analysis of variance was used to assess the significant differences between viral loads and between mRNA levels of genes. Tukey’s Multiple Comparison Test of post hoc analysis was used to further compare which pairs were significantly different. However, GraphPad Prism 5 did not show actual p values in this test but only indicated whether P < 0.05 or not. To assess the significant differences between four groups (10^0^, 10^2^, 10^4^ or 10^6^ pfu ) of JEV-infected mice, survival analysis was performed by Log-rank (Mantel-Cox) test.

## Competing interests

The authors declared they have no competing interests.

## Authors’ contributions

DH designed the study, KA, SS, DSS, MMNT and DH performed experiments, KA, KM and DH analyzed the data, and CCB and DH wrote the paper. All authors read and approved the final manuscript.

## References

[B1] Gubler JD Kuno G Markoff L Flaviviruses Knipe DM, Howley PM, Griffin DE, Lamb RA, Straus SE, Martin MA, Roizman B Fields Virology 2007 Philadelphia, PA: Lippincott Williams & Wilkins, a Wolters Kluwer Business 1153 1252

[B2] Erlanger TE Weiss S Keiser J Utzinger J Wiedenmayer K Past, present, and future of Japanese encephalitis Emerg Infect Dis 2009 15 1 1 7 19116041 10.3201/eid1501.080311 PMC2660690

[B3] Ghosh D Basu A Japanese encephalitis-a pathological and clinical perspective PLoS Negl Trop Dis 2009 3 9 e437 19787040 10.1371/journal.pntd.0000437 PMC2745699

[B4] Garcia-Tapia D Hassett DE Mitchell WJ Jr Johnson GC Kleiboeker SB West Nile virus encephalitis: sequential histopathological and immunological events in a murine model of infection J Neurovirol 2007 13 2 130 138 17505981 10.1080/13550280601187185

[B5] Albrecht P Pathogenesis of neurotropic arbovirus infections Curr Top Microbiol Immunol 1968 43 44 91 4970327 10.1007/978-3-642-46118-7_2

[B6] Burke SD Monath PT Knipe DM, Howley PM, Griffin DE, Lamb RA, Martin MA, Roizman B, Straus SE Flaviviruses Fields Virology 2001 Philadelphia, PA: Lippincott Williams & Wilkins 991 1041

[B7] Kimura T Sasaki M Okumura M Kim E Sawa H Flavivirus encephalitis: pathological aspects of mouse and other animal models Vet Pathol 2010 47 5 806 818 20551474 10.1177/0300985810372507

[B8] Larena M Lobigs M Croatia RD Immunobiology of Japanese encephalitis virus Flavivirus Encephalitis 2011 317 338 InTech

[B9] German AC Myint KS Mai NT Pomeroy I Phu NH Tzartos J Winter P Collett J Farrar J Barrett A Kipar A Esiri MM Solomon T A preliminary neuropathological study of Japanese encephalitis in humans and a mouse model Trans R Soc Trop Med Hyg 2006 100 12 1135 1145 16814333 10.1016/j.trstmh.2006.02.008

[B10] Hase T Dubois DR Summers PL Comparative study of mouse brains infected with Japanese encephalitis virus by intracerebral or intraperitoneal inoculation Int J Exp Pathol 1990 71 6 857 869 2177623 PMC2002376

[B11] Lennette EH Influence of age on the susceptibility of mice to infection with certain neurotropic viruses J Immunol 1944 49 175 191

[B12] Hayasaka D Nagata N Fujii Y Hasegawa H Sata T Suzuki R Gould EA Takashima I Koike S Mortality following peripheral infection with tick-borne encephalitis virus results from a combination of central nervous system pathology, systemic inflammatory and stress responses Virology 2009 390 1 139 150 19467556 10.1016/j.virol.2009.04.026

[B13] Hayasaka D Shirai K Aoki K Nagata N Simantini DS Kitaura K Takamatsu Y Gould E Suzuki R Morita K TNF-alpha acts as an immunoregulator in the mouse brain by reducing the incidence of severe disease following Japanese encephalitis virus infection PLoS One 2013 8 8 e71643 23940775 10.1371/journal.pone.0071643 PMC3733918

[B14] Larena M Regner M Lee E Lobigs M Pivotal role of antibody and subsidiary contribution of CD8+ T cells to recovery from infection in a murine model of Japanese encephalitis J Virol 2011 85 11 5446 5455 21450826 10.1128/JVI.02611-10 PMC3094953

[B15] Monath TP Guirakhoo F Nichols R Yoksan S Schrader R Murphy C Blum P Woodward S McCarthy K Mathis D Johnson C Bedford P Chimeric live, attenuated vaccine against Japanese encephalitis (ChimeriVax-JE): phase 2 clinical trials for safety and immunogenicity, effect of vaccine dose and schedule, and memory response to challenge with inactivated Japanese encephalitis antigen J Infect Dis 2003 188 8 1213 1230 14551893 10.1086/378356

[B16] Fujii Y Kitaura K Nakamichi K Takasaki T Suzuki R Kurane I Accumulation of T-cells with selected T-cell receptors in the brains of Japanese encephalitis virus-infected mice Jpn J Infect Dis 2008 61 1 40 48 18219133

[B17] Yoshikawa T Iwasaki T Ida-Hosonuma M Yoneyama M Fujita T Horie H Miyazawa M Abe S Simizu B Koike S Role of the alpha/beta interferon response in the acquisition of susceptibility to poliovirus by kidney cells in culture J Virol 2006 80 9 4313 4325 16611890 10.1128/JVI.80.9.4313-4325.2006 PMC1472025

[B18] Honda K Takaoka A Taniguchi T Type I interferon [corrected] gene induction by the interferon regulatory factor family of transcription factors Immunity 2006 25 3 349 360 16979567 10.1016/j.immuni.2006.08.009

[B19] Tamura T Yanai H Savitsky D Taniguchi T The IRF family transcription factors in immunity and oncogenesis Annu Rev Immunol 2008 26 535 584 18303999 10.1146/annurev.immunol.26.021607.090400

[B20] Loo YM Gale M Jr Immune signaling by RIG-I-like receptors Immunity 2011 34 5 680 692 21616437 10.1016/j.immuni.2011.05.003 PMC3177755

[B21] Taylor SS Haste NM Ghosh G PKR and eIF2alpha: integration of kinase dimerization, activation, and substrate docking Cell 2005 122 6 823 825 16179248 10.1016/j.cell.2005.09.007

[B22] Pfaller CK Li Z George CX Samuel CE Protein kinase PKR and RNA adenosine deaminase ADAR1: new roles for old players as modulators of the interferon response Curr Opin Immunol 2011 23 5 573 582 21924887 10.1016/j.coi.2011.08.009 PMC3190076

[B23] McAllister CS Samuel CE The RNA-activated protein kinase enhances the induction of interferon-beta and apoptosis mediated by cytoplasmic RNA sensors J Biol Chem 2009 284 3 1644 1651 19028691 10.1074/jbc.M807888200 PMC2615529

[B24] Hills SL Phillips DC Past, present, and future of Japanese encephalitis Emerg Infect Dis 2009 15 8 1333 19751614 10.3201/eid1508.090149 PMC2815973

[B25] Hayasaka D Ivanov L Leonova GN Goto A Yoshii K Mizutani T Kariwa H Takashima I Distribution and characterization of tick-borne encephalitis viruses from Siberia and far-eastern Asia J Gen Virol 2001 82 Pt 6 1319 1328 11369875 10.1099/0022-1317-82-6-1319

